# A Time-Course-Based Estimation of the Human Medial Olivocochlear Reflex Function Using Clicks

**DOI:** 10.3389/fnins.2021.746821

**Published:** 2021-10-28

**Authors:** Sriram Boothalingam, Shawn S. Goodman, Hilary MacCrae, Sumitrajit Dhar

**Affiliations:** ^1^Department of Communication Sciences and Disorders, University of Wisconsin-Madison, Madison, WI, United States; ^2^Waisman Center, University of Wisconsin-Madison, Madison, WI, United States; ^3^Department of Communication Sciences and Disorders, University of Iowa, Iowa City, IA, United States; ^4^Roxelyn and Richard Pepper Department of Communication Sciences and Disorders, Northwestern University, Evanston, IL, United States; ^5^Knowles Center, Northwestern University, Evanston, IL, United States

**Keywords:** medial olivocochlear reflex, middle ear muscle reflex, click-evoked otoacoustic emissions, time-course, kinetics

## Abstract

The auditory efferent system, especially the medial olivocochlear reflex (MOCR), is implicated in both typical auditory processing and in auditory disorders in animal models. Despite the significant strides in both basic and translational research on the MOCR, its clinical applicability remains under-utilized in humans due to the lack of a recommended clinical method. Conventional tests employ broadband noise in one ear while monitoring change in otoacoustic emissions (OAEs) in the other ear to index efferent activity. These methods, (1) can only assay the contralateral MOCR pathway and (2) are unable to extract the kinetics of the reflexes. We have developed a method that re-purposes the same OAE-evoking click-train to also concurrently elicit bilateral MOCR activity. Data from click-train presentations at 80 dB peSPL at 62.5 Hz in 13 young normal-hearing adults demonstrate the feasibility of our method. Mean MOCR magnitude (1.7 dB) and activation time-constant (0.2 s) are consistent with prior MOCR reports. The data also suggest several advantages of this method including, (1) the ability to monitor MEMR, (2) obtain both magnitude and kinetics (time constants) of the MOCR, (3) visual and statistical confirmation of MOCR activation.

## 1. Introduction

The auditory efferent system serves as a dynamic feedback mechanism through which the brain regulates afferent neural inputs. Such feedback control occurs at multiple stages in the auditory system and is thought to aid in automatic and attention-driven signal detection in noise (Winslow and Sachs, 1988; de Boer and Thornton, 2007; Delano et al., 2007; Mertes et al., 2019) and protection of peripheral sensory cells from acoustic overexposure (Galambos and Rupert, 1959; Borg et al., 1983; Liberman, 1990; Walsh et al., 1998; Rajan, 2000; Lauer and May, 2011; Liberman et al., 2014; Boero et al., 2018). The efferent system is also implicated in disorders such as auditory neuropathy where its function is diminished (Hood et al., 2003; Valero et al., 2018), and in tinnitus and hyperacusis where it is hyperactive (Knudson et al., 2014; Wilson et al., 2017; Wojtczak et al., 2017). The most caudal and widely investigated of these feedback mechanisms are the medial olivocochlear reflex (MOCR) and the middle ear muscle reflex (MEMR). The MOCR inhibits cochlear amplification by limiting outer hair cell (OHC) motility (Siegel and Kim, 1982; Guinan and Gifford, 1988) and the MEMR reduces signal transfer through the middle ear by stiffening the ossicular chain (Borg et al., 1983; Liberman and Guinan, 1998). For decades, the MEMR has been clinically used to differentiate cochlear vs. neural pathologies (Jerger et al., 1974; Borg et al., 1983; Berlin et al., 2005). However, a reliable test of the MOCR currently does not exist. To fill this longstanding gap, here we describe a time-course and click-evoked otoacoustic emission (CEOAE)-based method that has the potential to serve as a simple and efficient test of efferent modulation of cochlear function.

**Figure 1:**
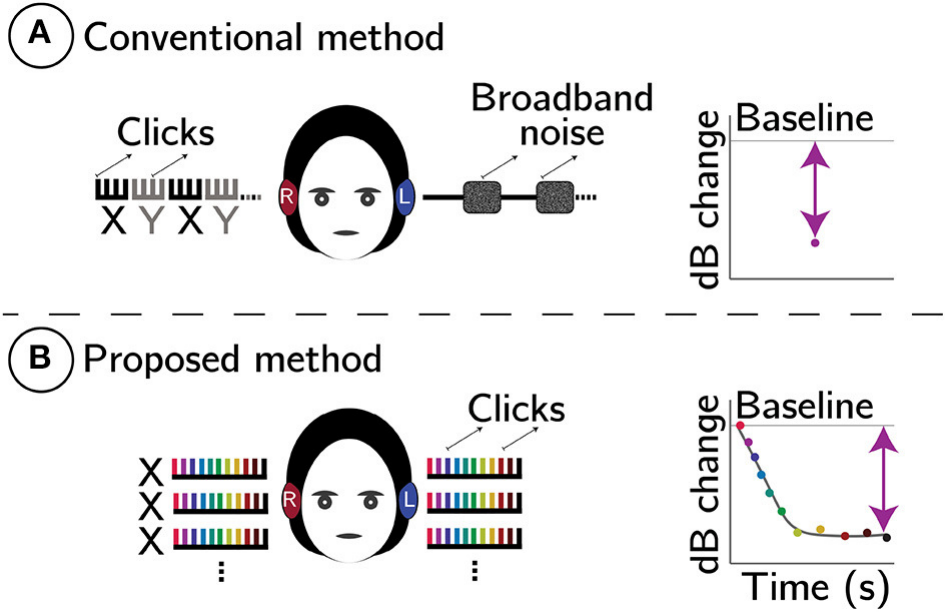
Schematic comparison between conventional **(A)** and proposed **(B)** MOCR methods. In both panels, comb-like structures represent click trains. In **(A)**, X (black) are baseline CEOAEs without, and Y (gray) are with contralateral noise elicitor, respectively. Plots on the right are predicted change in CEOAE level (dB). In **(B)**, whole click-trains (X) are averaged and CEOAE inhibition is estimated using a two-term exponential fit to the change in CEOAE level, Δ, as a function of time. The line-fit also provides reflex kinetics (time-constants).

Given that the MOC fibers directly innervate the OHCs (Warr and Guinan, 1979), OAEs provide a non-invasive means to investigate the influence of the MOCR on the OHCs (Guinan, 2006, 2014; Lopez-Poveda, 2018). Typically, a change in the OAE amplitude is monitored in the ipsilateral ear in response to MOCR activation in the contralateral ear with broadband noise (BBN; referred henceforth as the conventional method, see Figure 1A). While this method is convenient, it can be improved further in several ways:

(1) Conventional methods only test the contralateral pathway of the bilateral MOCR reflex system. Because BBN is presented in the contralateral ear to elicit the MOCR, no meaningful estimate of the MOCR is possible in this ear. If the ipsilateral or bilateral MOCR were to be estimated, forward masking techniques (Berlin et al., 1995; Boothalingam et al., 2018) or notched spectrum-noise methods (Backus and Guinan, 2006) must be employed. However, forward masking methods are time onerous and only capture the decaying segment of the MOCR (Backus and Guinan, 2006) and notched-spectrum-noise methods are not conducive for all types of OAEs (e.g., clicks). Estimating the ipsilateral and bilateral MOCR activity independently as well as in combination would simply provide a reductionist as well as holistic examination of the MOCR system (Guinan, 2014).
(2) In the conventional method, the stimulus itself can inadvertently activate the ipsilateral MOCR to unknown degrees, introducing uncertainties in MOCR magnitude estimation (Guinan et al., 2003; Boothalingam and Purcell, 2015; Boothalingam et al., 2018).
(3) Multiple MOCR studies have reported on the rather sub-par test-retest reliability of the conventional method (Mishra and Lutman, 2013; Stuart and Cobb, 2015; Mertes and Leek, 2016; Killan et al., 2017). This issue may, in part, be due to the reliance of the conventional method on “block averaging” (XYXY in Figure 1A) which is vulnerable to participant-related artifacts (e.g., change in middle ear pressure over time, probe drifts, etc.). The vulnerability comes from the temporal separation of the OAEs measured with and without the contralateral elicitor. This separation ranges between seconds to minutes across studies. Longer the gap between conditions, higher the risk of spurious changes in OAE level and probe drifts (Goodman et al., 2013).
(4) As illustrated in Figure 1A, conventional methods reduce the MOCR inhibition to a single data point in time, essentially decimating any data on reflex kinetics. That is, the evolution of the reflex over time cannot be gleaned from these methods.

As such, there is persistent uncertainty as to whether the change in OAE is due to the MOCR, participant-related artifact, or a systematic shift in measurement parameters.

Here, we propose a method that re-purposes the OAE-evoking clicks to also elicit and monitor MOCR activity. Click parameters used in this approach were identified in our prior work to optimally activate the MOCR while allowing adequate time for extracting CEOAEs (Boothalingam and Purcell, 2015; Boothalingam et al., 2018). Here, we extend the previous findings by employing these parameters [level: 80 dB peak-to-peak equivalent (pe)SPL; rate: 62.5 Hz] to test whether MOCR magnitude and time-constants can be extracted with either ear (left/right) and bilateral stimulation. This method is illustrated in Figure 1B.

The proposed method overcomes the limitations of the conventional method in the following ways:

(1)By using the same clicks that evoke CEOAEs to activate the MOCR, we relinquish the need to use a separate noise elicitor in the contralateral ear. This freedom from noise elicitor allows us to measure CEOAEs in both ears simultaneously and, consequentially, index the bilateral MOCR activity. Ironically, the limitation of this method is that the contralateral pathway cannot be evaluated separately.
(2) Because the proposed method does not require separate with- and without-noise conditions, conventional block-averaging is not necessary. As illustrated in Figure 1B, the entire click-train is averaged, which includes both the baseline (time zero) and the subsequent change in CEOAE over time. This short duration, unlike conventional methods where the with- and without-noise conditions are temporally separated, is predicted to minimize the undue influence of participant-related artifacts and/or probe drifts.
(3) In contrast to the uncertainty in the measured change in OAE being attributed to the MOCR in conventional methods, the well-established time-course of the MOCR—a two-term exponential with fast and slow time constants (Liberman et al., 1996; James et al., 2005; Backus and Guinan, 2006)—is exploited in the proposed method to determine if the change in the OAE is indeed due to the MOCR. While the time-course information can be obtained using contralateral noise in a conventional paradigm, it is time prohibitive and can only be obtained for one ear at a time.

**Figure 2:**
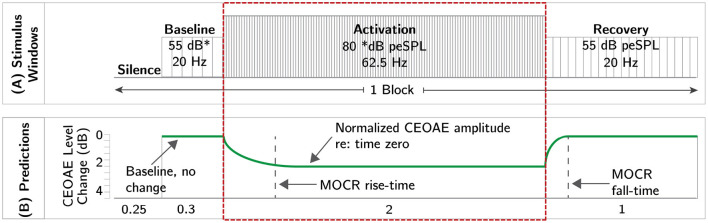
Schematic of the experimental paradigm. Panel **(A)** illustrates the temporal order of different windows presented in the experiment. Panel **(B)** illustrates the predicted change in CEOAE level across different windows (green curves). The activation window, highlighted by the dotted red rectangle, is the same as the click paradigm described in Figure 1B. The duration of each click window are provided at the bottom of panel **(B)**.

We hypothesize that click-train averaging will preserve the time-course of the MOCR allowing for the extraction of MOCR magnitude and kinetics. The predicted change in CEOAE level is grossly illustrated in Figure 1B and more specifically in Figure 2B. The overarching goal of this work is to demonstrate the feasibility of the proposed CEOAE and time-course-based test of the MOCR.

## 2. Methods

### 2.1. Participants

A total of 17 participants in the age range 18–30 yrs were recruited for the study in compliance with the guidelines of the Northwestern University Institutional Review Board and were compensated monetarily for their participation. All participants had an unremarkable otoscopic examination, hearing thresholds ≤ 20 dB HL between 0.25 and 8 kHz (Audio Traveler AA220, Interacoustics, Assens, Denmark) and clinically normal tympanometry (GSI TympStar, Grason-Stadler Inc., Eden Prairie, MN). Participants were also required to have measurable distortion product OAE (DPOAE) (2f1-f2; f2/f1 = 1.22; L1/L2 = 55/40 dB SPL) with signal-to-noise ratio >6 dB between 0.5 and 6 kHz. Middle ear muscle reflex (MEMR) thresholds evoked using clicks presented at 100 Hz were monitored using a 226 Hz tonal probe (GSI TympStar, Grason-Stadler Inc., Eden Prairie, MN) and were required to be >75 dB HL for inclusion. Two participants were rejected due to this inclusion criterion. One more participant was rejected due to the presence of more than five spontaneous OAEs (SOAEs) that were ~3 dB above the noise floor (Boothalingam et al., 2018). SOAEs influence CEOAE-based MOCR estimation as well as MEMR. Therefore, it is prudent to limit SOAE contributions. The final sample size was 14 [Mean age = 21.1; standard deviation (SD) = 1.9 yrs].

All testing was completed inside a double-walled sound booth where participants sat in a comfortable recliner for the duration of the experiment. During the experiment, participants watched a silent closed-captioned movie of their choice. Participants were encouraged to relax, not swallow, and stay awake during periods of stimulus presentation. Breaks were provided every 8 min, during which participants were encouraged to stretch, drink water, and do other noisy activities that were discouraged during recording periods. Throughout the experiment, OAE probes in both ears of participants were sealed using earmold putty to avoid slippage/drifts. The entire experiment took roughly 3 hrs to complete per participant. All testing was completed in a single session.

### 2.2. Stimulus Generation

All stimuli were digitally generated in Matlab (v2016b; Mathworks, MA, USA) at a sampling rate of 96 kHz and a bit depth of 24. Similar to Boothalingam et al. (2018), clicks were generated in the frequency domain using a recursive exponential filter (Zweig and Shera, 1995; Charaziak et al., 2020) for band-limiting the click between 0.8 and 6 kHz (~108 μs long). Bandpass clicks were used to focus the stimulus energy in the frequency regions where the MOCR is most prominent (Lilaonitkul and Guinan, 2012; Zhao and Dhar, 2012). In addition, bandpass clicks produced less loudspeaker ringing in our set-up compared to a single sample impulse.

### 2.3. Instrumentation and Calibration

Instrumentation was similar to that described in Boothalingam et al. (2018). Briefly, signal delivery and data acquisition were controlled through an iMac computer (Apple, CA, USA) running Auditory Research Lab Audio Software (ARLas v4.2017 Goodman, 2017) on Matlab at 96 kHz. Digital-to-analog and analog-to-digital conversions were handled by an external sound card (Fireface UCX; RME, Germany) connected to the iMac via Firewire. Signals were delivered bilaterally via two ER2 insert receivers coupled to two separate ER10B+ probe assemblies (Etymotic Research, IL, USA). The second port in the ER10B+ probe was coupled to identical dummy loudspeakers bilaterally. Ear canal pressure was registered and amplified (+20 dB) by the ER10B+ probe microphone and pre-amplifier, respectively.

The root-mean-square (RMS) level of BBN was calibrated in a Zwislocki ear simulator. Click levels were also first calibrated in a Zwislocki ear simulator where its peak-to-peak amplitude was matched with a 1 kHz sine tone. In addition, an in-ear calibration was performed in each participant. In this approach, a sample of clicks were played in the participant's ears before the start of every condition and any deviations from the expected peSPL at the probe-tip were corrected.

### 2.4. Experimental Paradigm

A schematic of the experimental paradigm is illustrated in Figure 2. The hierarchy of terminologies is as follows. Each click presentation and the silent duration which follows until the onset of the next click is an “epoch.” Therefore, epoch durations vary with click rate. Clicks with different levels and rates served different purposes and were grouped into “windows.” The difference in column width in each window in Figure 2A represents the epoch duration and the height represents the click level. Four different “windows” made up a single “block.” Blocks were repeated 500 times. The “silence” window (250 ms), where no stimulus was presented, allowed the MOCR to return to baseline functioning (Backus and Guinan, 2006). In the “baseline” (300 ms), low-level (55 dB peSPL) and slow-rate (20 Hz) clicks that are known to not activate the MOCR or the MEMR were presented (Boothalingam and Purcell, 2015; Boothalingam et al., 2018). CEOAEs in the baseline window served as confirmation for MOCR activity starting from the baseline no activity in the “activation” window where higher level (80 dB peSPL) and faster rate (62.5 Hz) clicks were presented for 2 s. The click level and rate used in this window are based on our prior work that demonstrated robust MOCR activation with little-to-no evidence of MEMR activation (Boothalingam and Purcell, 2015; Boothalingam et al., 2018). Finally, the same slow rate and low level clicks from the baseline were presented again for 1 s in the “recovery” window to capture the MOCR decay. The same paradigm was presented in three lateralities which included two unilateral stimulations, left- and right-only stimulation, and one bilateral stimulation. However, for the sake of brevity, ear canal recordings from only one ear from bilateral stimulation is discussed in this paper. This includes an equal number (7) of right and left ears.

Note that because the click levels and rates are different across windows, the evoked OAEs cannot be considered as a continuous function over time. However, despite the rate/level differences between activation and recovery windows, the elicited MOCR activity is considered a continuous function of time across these two windows. This is because it is the MOCR elicited by the click-train in the activation window that is being captured in the recovery window. Slowing the click rate and lowering the click level is essential in this process because continuing the same high rate and level from the activation window will not allow the MOCR to decay.

### 2.5. CEOAE Extraction

Raw microphone pressure recordings were processed offline using custom scripts written in Matlab. First, all pressure recordings were bandpass filtered between 0.8 and 4.2 kHz—close to the bandpass frequency of the click stimulus. Next, any epochs that had an RMS amplitude >2.25 times the interquartile range (within-participant) were rejected as containing artifacts. Overall, less than 10% of the data were rejected across included participants.

All MOCR analyses were conducted on CEOAEs time-windowed between 4.5 and 15 ms relative to time zero. Time zero was defined as the location of the peak of the click stimulus. Hann ramps (1 ms long) were applied at the start and end of the CEOAE waveform. Prior to any analysis, epochs within the different stimulus windows were sub-averaged by a factor of 2. For instance, in the 125 clicks (62.5 Hz × 2 s) recorded per block in the activation window, adjacent epochs were averaged. This sub-averaging, while reducing the resolution of the time-course by a factor of 2 (32 ms instead of 16 ms in the activation window), allowed for estimation of CEOAEs at each time point from 1,000 epochs [500 repetitions × 2; Boothalingam and Goodman, 2021]. This step allowed us to reduce test times by half while still maintaining the quality of the recorded CEOAEs. The 32 ms resolution is smaller than the rise- and fall-time of the MOCR (Kim et al., 2001; Backus and Guinan, 2006). Therefore, this sub-averaging should not affect the quality of the time constants obtained.

Next, within each epoch, CEOAEs were considered in the time-frequency domain to more precisely extract the signal of interest. A time-frequency representation of the OAE was constructed using a bank of overlapping gammatone filters with center frequencies between 0.8 and 4.2 kHz (Goodman et al., 2021). The filters were based on models of human auditory filters (Glasberg and Moore, 1990). Stimulus frequency (SF) OAE-based delays (Shera et al., 2002, 2008) were used to time window the filtered waveforms so as to only include CEOAEs within the expected delays (± 20%) at each frequency. After time windowing, the filtered waveforms were added back together to yield composite waveforms. This approach improves signal-to-noise ratio because noise energy is excluded from temporal regions where no OAEs are expected in each filer band. As such, the steps described herein allowed for the extraction of time (within each epoch) and frequency (specific bands of interest)-based CEOAEs at each time point in the respective windows.

Following extraction, the CEOAEs within each frequency band were averaged across time (within each epoch). The averaging process also included taking the energy-weighted average within each frequency band. That is, the spectral energy, which is the square of the pressure magnitude at each Fourier frequency, was used as weights for computing the weighted mean across frequency. This reduced the contribution of frequencies with small OAE magnitude relative to frequencies with higher OAE magnitude. Averaging was performed separately for each point in the time series, i.e., every 32 ms. This process reduced the time-frequency representation of the CEOAE to 7 spectral magnitudes corresponding to the passband frequencies of a bank of third-octave filters with nominal center frequencies 1, 1.2, 1.6, 2, 2.5, 3.2, and 4 kHz, where the MOCR effects are predominant (Lilaonitkul and Guinan, 2012; Zhao and Dhar, 2012). The CEOAE magnitude at each frequency and time point was calculated as the magnitude of the mean across the 500 repetitions. Within each frequency band, noise floor was estimated as the standard error of the mean of the CEOAE spectra (Goodman et al., 2009). An SNR criterion of 12 dB was imposed for CEOAEs at each frequency to be included. Frequencies, typically spectral notches, where the SNR was lower than 12 dB were not included in the averaging process.

### 2.6. Time-Course Analysis for MOCR Estimation

For each frequency band, the magnitude in Pascals at each point in the time series was divided by the magnitude in Pascals at the first time point for the activation window and the last time point for the recovery windows. Recall that each time point is an average of 1,000 click epochs across 0-32 ms, i.e., two consecutive epochs in time repeated 500 times. This use of within-window baseline is one of the strengths of the proposed method for MOCR estimation as it does not require a separate baseline measurement. Referred to hereafter as Δ, this metric of relative change was then expressed in dB. This final step allowed for easier visualization of the change in CEOAE over time across frequencies and participants. As illustrated in Figure 2B, no change in the CEOAE over time can be imagined as a straight line at 0 dB. A negative Δ, i.e., reduction in CEOAE magnitude, would indicate potential MOCR activation.

MOCR activation was quantified as the change in Δ at 2 s, the final time point in the activation window, and termed Δ_*max*_. The change and the associated rise- and fall-times, were estimated using a two-term exponential line fit to the CEOAE data, similar to the implementation of this method for MEMR estimation (Boothalingam and Goodman, 2021) and based on DPOAE rapid adaptation (Liberman et al., 1996; Kim et al., 2001; Srinivasan et al., 2012). A two-term exponential fit has previously been shown to provide a good estimate of the MOCR as the MOCR activation works on at least two time scales: fast and slow (Sridhar et al., 1995; Liberman et al., 1996; Kim et al., 2001; Backus and Guinan, 2006). The two-term exponential was of the form:


(1) $$\begin{array}{r} f\left(t\right)=C+m_{f}*e^{\left(-t/tau_{f}\right)}+m_{s}*e^{\left(-t/tau_{s}\right)}, \end{array}$$

where *f* is the fit as a function of time, *t*. The variables *m*_*f*_ and *m*_*s*_ are the magnitude of the fast and slow components of the fits, respectively. The variables *tau*_*f*_ and *tau*_*s*_ are the fast and slow time-constants, respectively. *C* is a constant term representing offset along the y axis. To determine if the Δ approximated by the two-term exponential fit is statistically significant, we employed a permutation-based implementation of the Heller-Heller-Gorfine (HHG; Heller et al., 2013) test as described by Boothalingam and Goodman (2021) for MEMR estimation. Briefly, the fit and Δ were compared as two vectors hypothesized to have no association, i.e., at least one of the two vectors, more likely the Δ, changes randomly over time. Significance of the comparison (*p*-value) was obtained by generating confidence intervals from bootstrapping the HHG test 1,000 times. Because seven such tests were conducted for any given laterality/window, the *p*-values were corrected for performing multiple comparisons using Bonferroni correction. A significant fit was considered as MOCR activation. Our pilot data indicated a lack of MOCR activation in the baseline window. Therefore, we regressed Δ in the baseline window against time using simple linear regression to test for any systematic change over time.

### 2.7. Test for MEMR Activation

The presence of MEMR may influence MOCR activation, and therefore the recorded responses must be carefully examined. Activation of the MEMR alters the impedance characteristics of the middle ear. Because impedance is frequency dependent, it is important to recognize that different frequencies are affected differently. At frequencies below ~0.8 kHz and above ~1.5 kHz, there is an increase in stimulus reflectance whereas there is a reduction in reflectance between ~0.8 and 1.5 kHz (Feeney and Keefe, 1999; Feeney et al., 2017; Boothalingam and Goodman, 2021). We used the same time-course method used for the MOCR, except the use of time-frequency analysis, and as described by Boothalingam and Goodman (2021), to determine MEMR activation in all seven frequency bands. The difference between the MOCR and the MEMR analyses is that the stimulus waveform (0–4 ms) was analyzed to determine the presence of the MEMR while the CEOAE waveform was analyzed to determine the presence of the MOCR.

## 3. Results

### 3.1. MEMR Activation Has Minimal Effect on MOCR Magnitude

**Figure 3:**
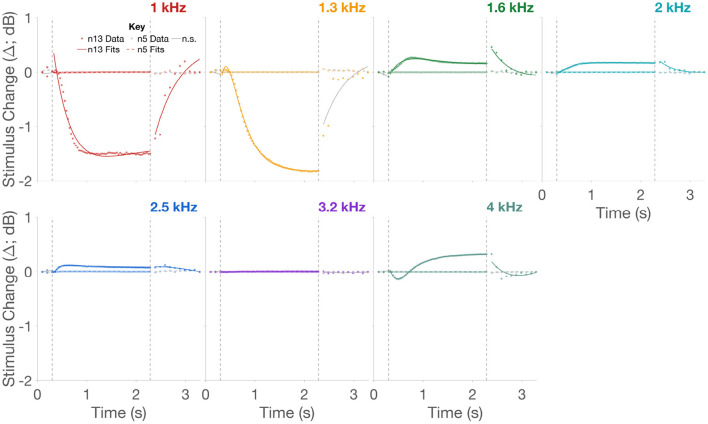
Stimulus change over time in two representative participants. Panels separate 1/3rd octave-band frequencies. Time-course data for the two participants, n5 and n13, are shown as squares and circles, respectively. Fits to the data are shown as dashed and unbroken lines, respectively. Colors represent the different frequencies. Fits lines in the respective color are statistically significant while fit lines in gray are not. The two vertical dashed lines in panels indicate the temporal separation between the three windows.

While our previous broadband, and arguably less sensitive, approach to MEMR detection (Boothalingam et al., 2018) suggested that 80 dB peSPL clicks presented at 62.5 Hz should not significantly activate the MEMR, studying stimulus Δ in narrow bands of frequencies in the present approach shows MEMR activation in 100% of the participants for all three lateralities. Representative data for bilateral stimulation from two participants, one with large and one with small MEMR activation, are presented in Figure 3. Notice that although both participants demonstrate statistically significant stimulus Δ in the activation window, their Δ_*max*_ are vastly different, especially at the lower frequencies. For instance, Δ_*max*_ in the 1.3 kHz band for n13 is 1.8 dB compared to 0.0026 dB for n5. That is, the Δ_*max*_ of MEMR for n13 is ~700 times larger than that of n5. A natural question then is: do all MEMR activations necessitate influence on MOCR Δ_*max*_? To answer this question, we computed Pearson correlation coefficients between MOCR and MEMR Δ_*max*_ at and across all seven bands of frequencies.

**Figure 4:**
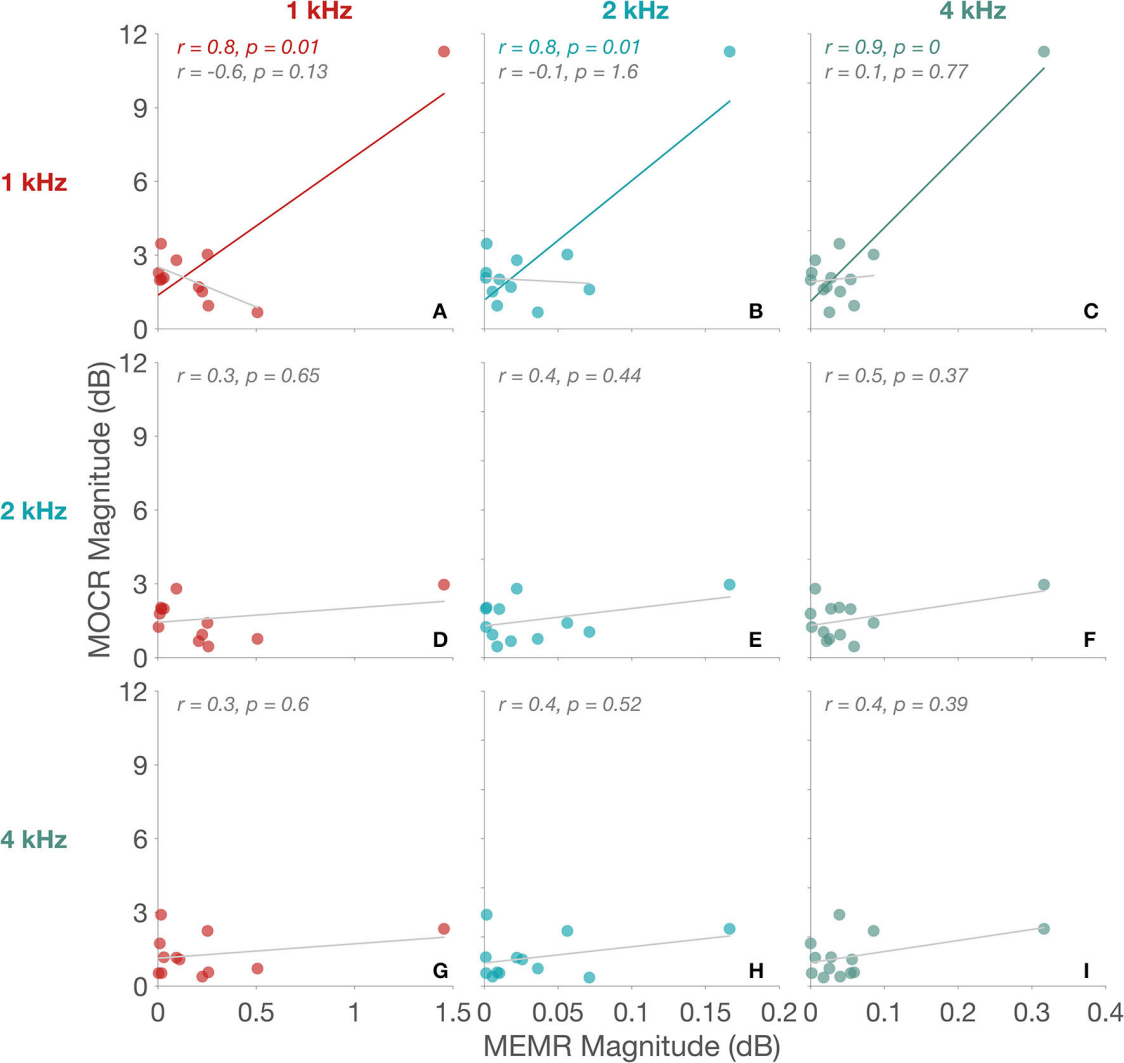
MEMR vs. MOCR. Panels **(A96I)** separate 1/3rd octave-band frequencies. MEMR magnitude are along the x-axis and MOCR magnitude are along the y-axis in all panels. MEMR frequencies are differentiated in panel columns and MOCR frequencies in panel rows. Comparison frequencies in each panel are the two frequencies intersecting the specific panel [e.g., panel **(A)** compares both MOCR and MEMR magnitude at 1 kHz]. Significant fits are indicated by frequency specific colors and non-significant fits are in gray. Corresponding Pearson correlation coefficient (*r*) and the *p*-value are provided at the top of each panel.

Scatter plots of absolute MOCR Δ_*max*_, i.e., MOCR magnitude vs. absolute MEMR Δ_*max*_, i.e., MEMR magnitude, for 1, 2, and 4 kHz bands are plotted in Figure 4. Only three of the seven frequencies are shown for brevity. As seen in the scatter plots, MEMR magnitude only correlates with MOCR magnitude when the outlier (n13) is included. Despite including the outlier, correlations were only significant for MOCR magnitude at 1 and 1.3 (not shown) kHz. Revaluation without the outlier did not produce any significant correlations even before correcting alpha for performing multiple comparisons using the False Discovery Rate (FDR) method (Benjamini and Hochberg, 1995). Evidently, the large change in stimulus in this particular participant, likely due to MEMR activation, has had an impact on their MOCR magnitude. As a group, however, the lack of correlations between MEMR and MOCR magnitude suggest that small changes in stimulus level, even if they are statistically significant, do not significantly affect MOCR estimates, at least in the 1–4 kHz frequency range. Therefore, only the n13 participant data were excluded from all remaining analyses. As such, the changes in CEOAE magnitude reported here are likely predominantly driven by the MOCR, not the MEMR.

### 3.2. Clicks Elicit Robust CEOAE Inhibition

**Figure 5:**
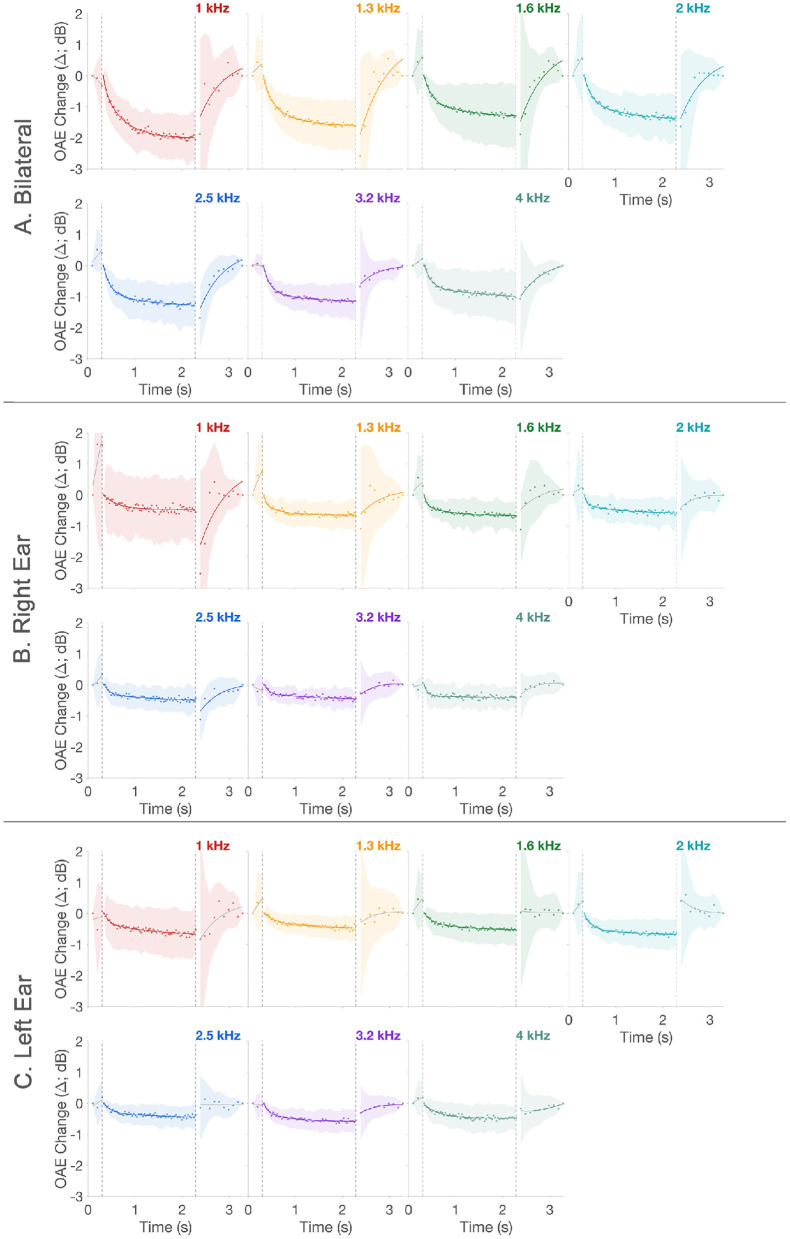
CEOAE change over time. Panels **(A–C)** show bilateral, right, and left ear mean time-course data, respectively. In all panels, scatter plot is the mean CEOAE change (Δ) across all 13 participants. The lines are statistical model fit to the data, linear (baseline) and two-term exponential (activation and recovery). Shaded region around the data represents ± 1SD around the mean. Colors represent the different frequencies. Fits lines in the respective color are statistically significant while fit lines in gray are not. The two vertical dashed lines in panels indicate the temporal separation between the three windows.

**Figure 6:**
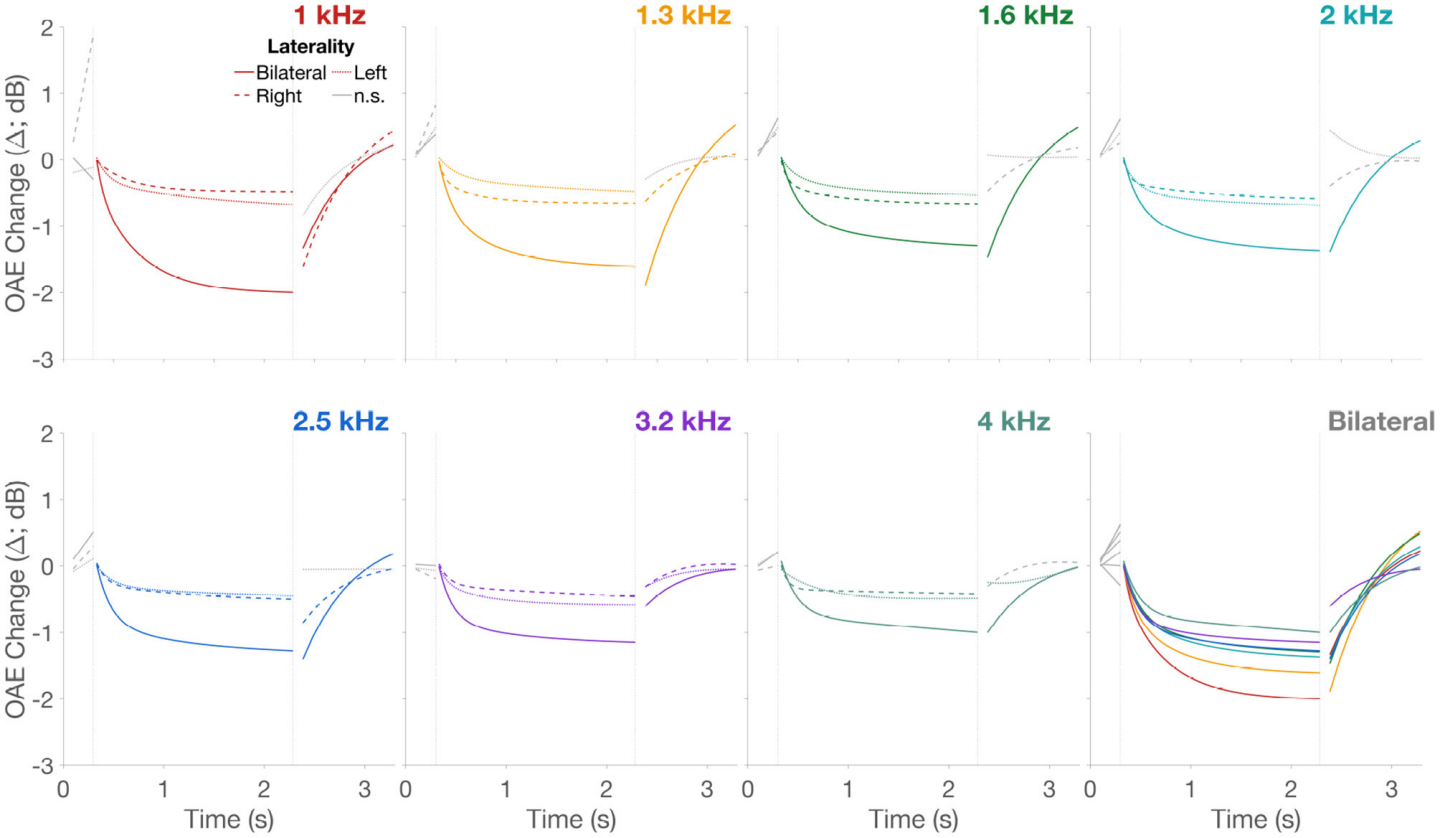
CEOAE change over time. Only the fits from Figure 5 are plotted to allow direct comparison across the three lateralities. Unbroken lines are bilateral, dashed lines are right, and dotted lines are left ear stimulation, respectively. Panels are separated by frequency in addition to the different colors for the 7 frequencies. Lines in gray are non-significant fits. Fits across all frequencies, only for the bilateral stimulation, is presented in the last panel “Bilateral” to allow direct visual comparison of CEOAE Δ as a function of frequency.

Mean Δ for all three windows across frequencies and lateralities are shown in Figure 5. As predicted in Figure 2B, there is no significant activity in the baseline window. This is followed by a significant Δ in the activation window, and finally the Δ returns to baseline in the recovery window. That is, clicks presented at 80 dB peSPL and 62.5 Hz (activation window) produced a significant inhibition of CEOAEs over the 2 s period in all three lateralities: right, left, and bilateral stimulation. When averaged across the seven frequencies, 91.2% of the two-term exponential fits (from a total of 91; 13 participants x 7 frequencies) were significant in the activation window for bilateral stimulation compared to 27.5% in the recovery window. The lower number of significant fits in the recovery window is likely due to lower click level (55 dB peSPL) and coarser time resolution (100 ms). For the right and left ear-only stimulation, the number of significant MOCR activations were lower at 58.2 and 71.4%, respectively. For a better comparison across lateralities, only the fits are presented in Figure 6. Similarly, for the recovery window, the percentage of significant fits to data for the right and left ear-only stimulation were also lower at 10.9 and 20.9%, respectively. No fits (0%) in the baseline window were significant for any of the three lateralities. Note that the mean Δ and all further analyses on MOCR/MEMR Δ_*max*_ and time constants were conducted only on data with significant fits to Δ. Due to the small number of significant fits in the recovery window and no significant fits in the baseline no further inferential statistics were conducted for data from these two windows.

**Figure 7:**
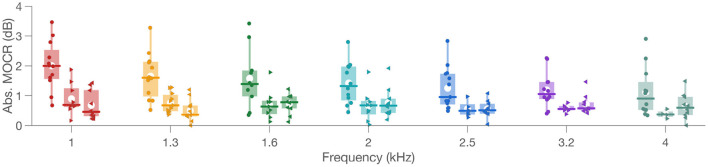
MOCR magnitude. Box plots show individual MOCR magnitude, i.e., absolute Δ_*max*_, as filled colored shapes at respective frequencies along the x-axis. Circle is bilateral, right-pointing triangle is right ear, and left-pointing triangle is for left ear data. Colors represent frequency in the x axis. In the box plots, the box represents the interquartile range, white circle is the mean, vertical line is the data range, and the horizontal line is the median.

MOCR Δ_*max*_ in the activation window extracted from fits to Δ are plotted as box plots in Figure 7. Two crucial observations can be made from Figures 5–7. (1) As expected based on binaural integration, bilateral stimulation produced larger, more than twice the MOCR inhibition (1.69 ± 1.2 dB; ± 1SD) relative to right (0.61 ± 0.4 dB) and left (0.62 ± 0.4 dB) ear-only stimulation. This is consistent with several prior reports (Berlin et al., 1995; Backus and Guinan, 2007; Lilaonitkul and Guinan, 2009a; Boothalingam et al., 2018, 2019). (2) At least for the bilateral stimulation, not all frequencies (Figure 6; Bilateral Panel) appear to be inhibited to the same extent. The largest mean inhibition is observed at the lower frequencies with inhibition progressively getting smaller. This is also consistent with prior work showing smaller MOCR activation above ~3 kHz (Goodman et al., 2013).

To study the data inferentially, a linear mixed-effects model was used. Laterality and frequency were fixed-effects while MOCR Δ_*max*_ was the dependent variable with random intercepts for each participant. An analysis of variance (ANOVA) of the model suggested a significant interaction between frequency and laterality [*F*_(12, 180)_ = 1.9, *p* = 0.04] with significant main effects of both laterality [*F*_(2, 180)_ = 35.3, *p* < 0.001] and frequency [*F*_(6, 180)_ = 5.9, *p* < 0.001]. *Post-hoc* pairwise comparisons for frequency differences within each laterality were conducted using *t*-tests corrected for multiple comparisons using the FDR method. Only three comparisons (out of 21) were statistically significant in the bilateral condition [1 vs. 2 kHz (*p* = 0.035); 1 vs. 2.5 kHz (*p* = 0.038); 1 vs. 4 kHz (*p* = 0.009)]. *Post-hocs* for the laterality effect suggested significantly larger MOCR Δ_*max*_ in the bilateral compared to both left [*t*(12) = −8.1; *p* < 0.001] and right [*t*(12) = −5.8; *p* < 0.001] ear stimulations, as expected. However, left and right ears were not significantly different [*t*(12) = −0.65; *p* = 0.53].

### 3.3. Click-Elicited CEOAE Inhibition Follows a Physiological Time-Course

**Figure 8:**
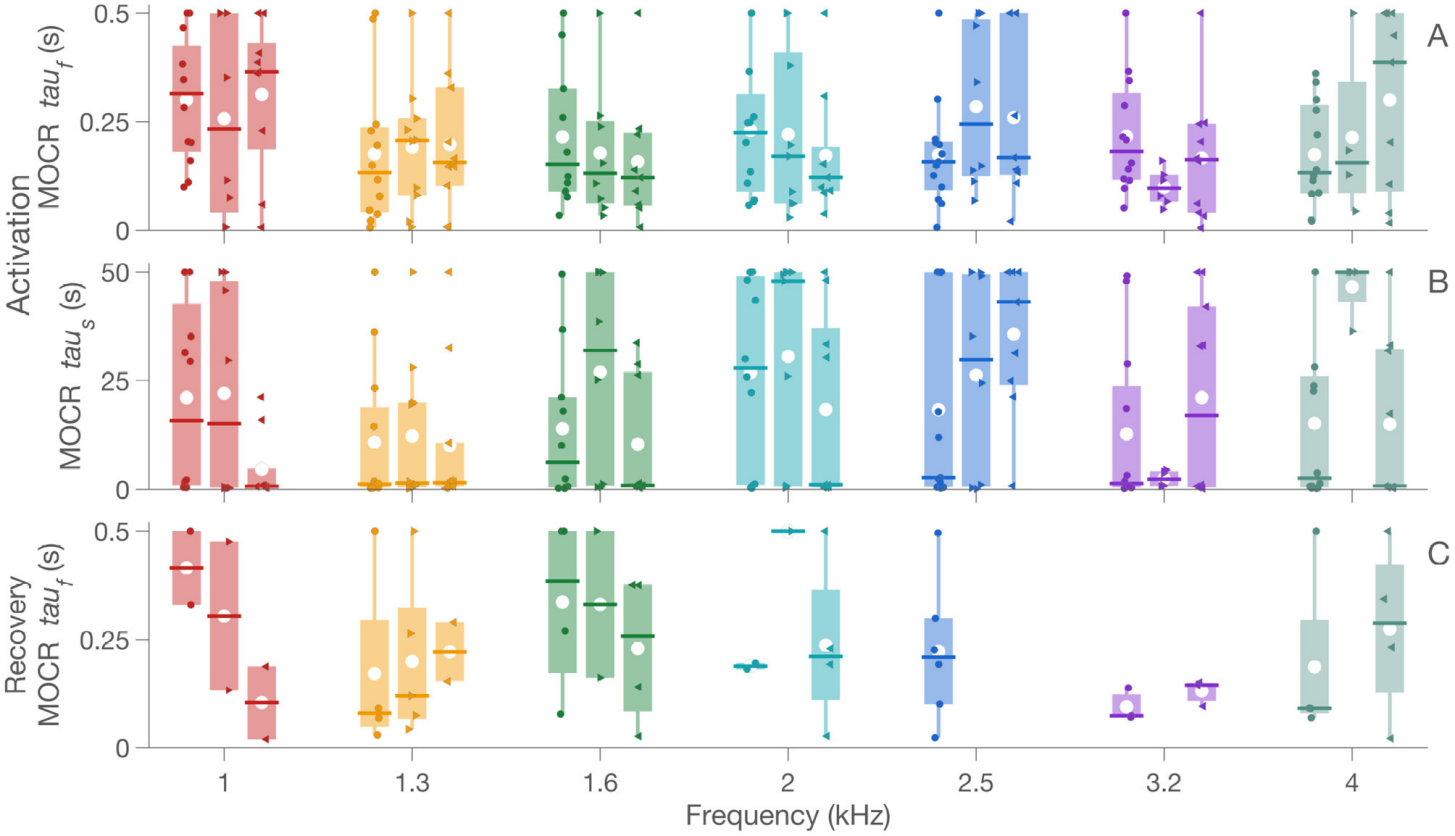
MOCR *tau*. Panels **(A,B)** show data for *tau*_*f*_ and *tau*_*s*_ in the activation window and panel **(C)** shows data for *tau*_*f*_ in the recovery window. Similar to Figure 7, box plots show individual MOCR *tau* as colored boxes at respective frequencies along the x-axis. Colored circle is bilateral, right-pointing triangle is right ear, and left-pointing triangle is for left ear data. In the box plots, the box represents the interquartile range, white circle is the mean, vertical line is the data range, and the horizontal line is the median.

Time constants derived from the two-term exponential fits are shown in Figure 8. The mean fast rise time (*tau*_*f*_) of the MOCR (averaged across frequencies) for the three lateralities were essentially the same: 0.22 ± 0.15, 0.21 ± 0.16, and 0.21 ± 0.17 s for bilateral, right, and left ear stimulation, respectively. These average values are consistent with the 0.28 ± 62 s reported by Backus and Guinan (2006). Unlike MOCR Δ_*max*_, there was no effect of laterality for the rise time *tau*_*f*_ [*F*_(2, 198)_ = 0.01, *p* = 0.99], frequency [*F*_(6, 198)_ = 1.0, *p* = 0.39], or their interaction [*F*_(12, 198)_ = 0.9, *p* = 0.46]. This result is also consistent with the findings of Backus and Guinan (2006) where they demonstrated the independence of MOCR time constants from elicitor level or laterality effects.

For the slow rise time, *tau*_*s*_, however, time constants for the three lateralities were slightly more variable: 16.8 ± 20, 23.8 ± 16.9, and 17 ± 17.5 s for bilateral, right, and left ear stimulation, respectively. Nonetheless, these values are also similar to those reported by Backus and Guinan (2006). The mixed-effects model suggested a significant interaction between laterality and frequency [*F*_(12, 198)_ = 2.2, *p* = 0.02] and a main effect of laterality [*F*_(2, 198)_ = 3.3, *p* = 0.04]. The fixed-effect of frequency was not significant [*F*_(6, 198)_ = 0.97, *p* = 0.44]. Because the main effects of frequency was not significant, data were collapsed across frequency to test for the effect of laterality. This *post-hoc* analysis, with *p*-values corrected for multiple comparisons using the FDR method, suggested no difference between right and left [*t*(12) = 1.1; *p* = 0.44], or left and bilateral [*t*(12) = −0.1; *p* = 0.92], or right and bilateral [*t*(12) = −1.5; *p* = 0.45].

The mean fall times (averaged across frequencies) were 0.22 ± 0.13, 0.33 ± 0.17, and 0.19 ± 0.14 s for bilateral, right, and left ear stimulation, respectively. These values are slightly longer than the 0.16 ± 0.5 s reported by Backus and Guinan (2006). Although no statistics were performed for the fall times (recovery window) due to the sparseness in the data, raw fall *tau*_*f*_ are shown in Figure 8. These values must be interpreted cautiously as only between 10 and 27% of the data produced significant fits.

To test whether the MOCR Δ_*max*_ at 2 s is statistically different from that at earlier times (1, 1.25, 1.5 s) we performed *t*-test, corrected for multiple comparisons using the FDR method. Four of the 7 frequencies (1, 1.3, 2, and 2.5 kHz; *p* <0.022) at 1 s was significantly different from that at 2 s. At 1.25 s, this number reduced slightly to 3 of 7 frequencies (1.3, 2, and 2.5 kHz; *p* <0.03). At, 1.5 s, only one of the 7 frequencies (2 kHz; *p* = 0.035) produced significantly different MOCR Δ_*max*_ than that at 2 s. This result suggests that click train duration between 1 and 1.5 should be sufficient to estimate the MOCR using the proposed approach.

## 4. Discussion

The results presented here provide evidence that clicks can be used to elicit and estimate MOCR activity simultaneously without the need for a contralateral noise elicitor.

### 4.1. MOCR Magnitude

The click level (80 dB peSPL) used in the present approach is higher in comparison to conventional MOCR methods (typical: 55–75 dB peSPL Veuillet et al., 1991; Hood et al., 1996; Goodman et al., 2013; Lewis, 2019). The 80 dB peSPL was chosen for its ability to elicit robust MOCR activity in a forward masking paradigm where the MOCR monitoring clicks were presented at 55 dB peSPL (Boothalingam et al., 2018). Higher click levels capture less MOCR activity as there is relatively less cochlear amplification for the MOCR to inhibit (Goodman et al., 2021). However, because our approach uses the same clicks to both elicit and monitor MOCR activity, the chosen click level must be able to play both roles. As such, the 80 dB peSPL is a compromise between (1) activating adequate MOCR activity (higher levels preferred), (2) capturing maximum possible MOCR activity using OAEs (lower levels preferred), and (3) avoiding MEMR activation (lower levels preferred). The mean MOCR Δ_*max*_ in the bilateral condition (1.69 dB) is commensurate with the 1–2 dB OAE inhibition typically reported in conventional noise-based studies for both contralateral and bilateral stimulations. While the bilateral stimulation in the present study would be expected to produce larger MOCR activation than the conventional contralateral noise stimulation paradigm, it should be noted that noise is a more potent elicitor than clicks (Veuillet et al., 1991; Guinan et al., 2003). It thus appears that the reduced potency of clicks in eliciting the MOCR is offset by bilateral stimulation. Similarly, the reduced potency of clicks in the current paradigm is offset by capturing the MOCR activity at its temporal peak unlike forward masked bilateral paradigms that although use noise but capture only the decaying portion of the MOCR. Finally, Lewis (2019) demonstrated that the larger OAE SNR counteracts the reduction in cochlear amplification at higher stimulus levels by allowing better detectability of MOCR activation. Therefore, levels around 80 dB peSPL seem ideal for the present approach. Taken together, it can be argued that our stimulus choice accomplishes both activation and monitoring of the MOCR similar to currently available methods.

The level that was optimal for bilateral stimulation did not elicit adequate MOCR activity in the unilateral conditions. The mean MOCR Δ_*max*_ for both right and left ears were <1 dB. This result is not unexpected based on the known physiology of the MOC neurons in the brainstem. Bilateral stimulation activates both ipsilateral and contralateral MOC neurons in addition to binaural MOC neurons (Liberman and Brown, 1986; Liberman, 1988). Evidently, there is a considerable increase in the number of neurons that are activated during bilateral stimulation. Furthermore, bilateral stimulation also allows for the capture of both crossed and uncrossed MOC fiber action in the cochlea. Therefore, bilateral stimulation not only elicits larger activity but also provides a complete picture of the MOCR function by activating all types of MOC neurons and pathways. There is also a considerable inter-species difference in the distribution of contralateral vs. ipsilateral MOC neurons. For instance, about 90% of the neurons respond to ipsilateral sound (Liberman and Brown, 1986) in cats, while about 50–55% respond to ipsilateral sound (Robertson and Gummer, 1985; Brown, 1989) in guinea pigs. Although this distribution is unknown in humans, it can be surmised from OAE-based studies that there may not be a large difference between ipsilateral and contralateral neuron count as they produce similar MOCR magnitude (Guinan, 2006). However, ipsilateral and contralateral stimulations do indeed produce varying degrees of activation when narrowband stimuli are employed, thought to be driven by unknown central processes rather than the MOC neurons themselves (Lilaonitkul and Guinan, 2009a).

The results of *post-hoc t*-tests for MOCR Δ_*max*_ corroborate the larger MOCR activation at around 1 kHz consistently reported in the literature (Lilaonitkul and Guinan, 2012; Zhao and Dhar, 2012). The smaller MOCR Δ_*max*_ in the unilateral stimulation presumably was not large enough to demonstrate such frequency effects. As such, these results indirectly highlight the importance of the size of the MOCR magnitude to reliably study the influence of experimental variables on MOCR activity (e.g., task difficulty, attention). If the MOCR Δ_*max*_ were not large enough to capture the effects of such variables, the presence of a potentially underlying effect may be rejected in error. Taken together, it does appear that bilateral stimulation is a better approach to study the function of the MOCR more completely and robustly. Because bilateral stimulation, using forward masking, in conventional noise elicitor-based methods capture only the decaying portion of the reflex, the proposed time-course-based method provides a feasible solution for both research and clinic. It should, however, be noted that despite capturing only the decaying portion of the reflex, noise elicitor-based forward masking paradigms do produce MOCR magnitude comparable to the current approach. This is likely due to noise elicitors being more potent than clicks (Veuillet et al., 1991; Guinan et al., 2003).

A byproduct of measuring ipsilateral and bilateral stimulation is the ability to study binaural interaction. The larger bilateral MOCR (1.69 dB) relative to the sum of right and left ear MOCR (0.61 + 0.62 = 1.23 dB) demonstrates “binaural interaction” reported in our prior work that used forward masking (Boothalingam et al., 2016). While measuring binaural integration was not one of the motivations of this study or approach, observing such well-known effects in our method provides confidence, that the CEOAE inhibition observed here is quite likely driven by MOCR activation. Another aspect of the MOCR that can be readily compared in this approach is the difference between left and right ears. This difference can be studied using unilateral or bilateral stimulation. For instance, the results of *post-hoc* tests between unilateral left/right ear stimulation suggested no difference between left and right ears. Although this is contrary to some studies (Khalfa et al., 1997; Morlet et al., 1999; Bidelman and Bhagat, 2015), others have reported similar results (Philibert et al., 1998; Xing and Gong, 2017). While animal (Gifford and Guinan, 1987) and human (Backus and Guinan, 2007; Lilaonitkul and Guinan, 2009a,b) studies have shown similarities and differences in the effects of crossed vs. uncrossed fibers on OAEs, the ear asymmetry in MOCR function remains unsettled. Using bilateral and unilateral stimulations in the present method, this question could be explored further in future studies.

### 4.2. MOCR Kinetics

#### 4.2.1. MOCR Activation

As predicted (Figures 1, 2A), the CEOAE Δ, i.e., the MOCR demonstrated a rise and a fall time. The two-term exponential fit approximated the data well, and as a result, we were able to extract the fast, *tau*_*f*_, and slow, *tau*_*s*_, rise time constants. Further, the lack of any significant fits in the baseline suggests that any Δ in the baseline window are likely a result of fluctuations in background noise over time. As seen in Figures 5, 6, these Δ can sometimes be larger than the Δ in the activation window. This is likely because CEOAEs elicited by the lower level clicks are less robust to background noise and therefore vary more over time. Crucially, these random fluctuations suggest that neither MOCR nor MEMR was active in the baseline window. Therefore, the MOCR and MEMR activity in the activation window can be surmised to have always started from the baseline, i.e., no activity, in every block. Alternatively, the non-significant Δ in the baseline window could be a result of relatively larger variance in this window, compared to the activation window, which may obscure small MOCR and MEMR effects.

The average *tau*_*f*_ across the three lateralities, 0.21 s, is commensurate with prior reports in humans (Backus and Guinan, 2006) as well as in animal models (Warren and Liberman, 1989; Liberman et al., 1996). Backus and Guinan (2006) also reported a slow time constant that was on the order of 10s of seconds and a medium time constant that was on the order of a few hundreds of milliseconds. An almost equal number of fits (participants × frequencies; 13 × 7) have *tau*_*s*_ of few hundreds of milliseconds (34%) and 10s of seconds (40%) in our data. It thus appears that both *tau*_*f*_ and *tau*_*s*_ in the present study may be mixtures of fast and medium, and medium and slow, time constants, respectively. We did not differentiate the *tau* into further smaller quantities as this was not the focus of the study. However, the corroboration with prior studies suggests that the time-course data reported here is of physiological, specifically of the MOCR, in origin. Prior reports have suggested an onset delay of the MOCR to be roughly between 25 and 60 ms (James et al., 2005; Backus and Guinan, 2006). The resolution of the time course in our approach, 32 ms, is too coarse to estimate such a short delay in the present study. Future studies that use clicks/tonebursts presented at faster rates (>62.5 Hz) when possible may be able to capture this detail with greater precision.

Also corroborating prior results (Backus and Guinan, 2006) were the lack of significant difference between lateralities or frequencies for the rise time *tau*_*f*_. This result suggests that despite the larger MOCR Δ_*max*_ for the bilateral stimulation, the time course of the MOCR effect on the periphery is the same as unilateral stimulation, at least during the fast onset phase. The slow rise time constant, *tau*_*s*_, however, was different between lateralties. This is largely driven by the higher *tau*_*s*_ registered in the right ears, the reasons for which are unclear. We randomized the probes between the right and left ears of participants, therefore this discrepancy is likely not measurement system related. It should be noted that the *tau* estimates reported here are partly dependent on the upper bound set to the two-term exponential fitting formula, 0.5 s for *tau*_*f*_ and 50 s for *tau*_*s*_. These upper bounds were set based on prior physiological data (Liberman et al., 1996). For some fits, the *tau* was essentially at this bound, likely due to a prolonged evolution of the reflex over time. This occurred in ~11% (participants × frequencies; 13 × 7) of the fits in bilateral (~12% in the right ear and ~13% in the left ear) stimulation despite these fits passing the HHG test. It is possible that such nuances affect *tau*_*s*_ differently from *tau*_*f*_. Further data are necessary to clarify such details.

Alternatively, with a higher degree of MOC activation (higher level) and/or better time resolution (faster rate), it is possible that *tau* estimation may be less variable. It is also possible that despite the average SNR for CEOAEs used in the fitting process being 26 dB, precise *tau* calculation may require even higher SNR. It should, however, be noted that it is not the *tau* estimation that is important for clinical translation of this approach. In fact, the usefulness of *tau* for the clinic is currently unknown. Instead, it is the statistically significant characteristic reduction in Δ, approximated by a two-term exponential function, that is critical to determine if the change in the CEOAE level is likely physiologically driven. This time-course is the biggest advantage of the present approach over conventional methods as a direct link between the Δ and the MOCR can be established with greater certainty.

#### 4.2.2. MOCR Steady-State

The MOCR Δ_*max*_ was estimated at the end of the 2-s activation window. In a majority of the participants, the Δ reached a steady-state earlier than 2 s. In a minority of the participants, it appeared to continue evolving, albeit gradually, even at the end of 2 s. If this method were to be translated to the clinic, the stimulus must be kept brief, 1 s or less. Comparisons between Δ_*max*_ estimated at 1, 1.25, 1.5, and 2 s suggest that a 1-second-long activation window along with a necessary silence period of 0.25–0.5 s, to allow MOCR to return to baseline, would be sufficient for MOCR estimation. This block duration would mean a test time of roughly 8 min. With further developments in signal processing, there is potential to reduce this test time further.

#### 4.2.3. MOCR Recovery

The CEOAE Δ_*max*_ return to baseline at the end of 1 s in the recovery window captures the decaying portion of MOCR activity. The lack of MOCR activation in the baseline window suggests that the 55 dB peSPL/20 Hz clicks in the recovery window should likely only capture the decay of the MOCR, (the fall time constant, *tau*_*f*_) and not activate any further MOCR activity. This recovery provides additional evidence that the clicks in the activation window did elicit the MOCR. However, the change in CEOAE level in the recovery window was not as robust as it was in the activation window. Unlike the rise time *tau*, we were unable to perform any statistics on the fall time *tau* due to the sparseness in the data. This is likely due to many reasons. (1) The poor time resolution in the recovery window relative to the activation window (100 vs. 32 ms). (2) Clicks evoking CEOAEs in the recovery window were much lower in level relative to the activation window (55 vs. 80 dB peSPL). (3) Lower click levels meant that the SNR was also lower relative to the activation window; 17.5 vs. 26 dB. These reasons likely rendered a larger proportion of the data in the recovery window unusable.

With these caveats in mind, The average fall time reported here (0.25 s; average across lateralties) is longer than that of the 0.16 s of Backus and Guinan (2006). This discrepancy could simply be due to the aforementioned caveats. Perhaps a higher level click and/or a faster click rate and/or longer averaging would capture this decay more robustly. However, higher click levels and faster click rates would also activate the MOCR and would not allow for the MOCR to decay. Therefore, one of the disadvantages of the proposed method is that the decay of the MOCR cannot be captured adequately in a clinically feasible time frame. However, it should be called to notice that the estimating the MOCR recovery for clinical purposes is not necessary. If the MOCR is activated, it will revert to its baseline activity given adequate time post stimulation. From the present study, Backus and Guinan (2006), and physiological data (Liberman, 1988; Liberman et al., 1996), it appears that 0.5 s should be sufficient for the MOCR to recover. This recovery time, along with the time to reach steady-state (1–1.5 s) is critical for designing future, more rapid, time-course-based MOCR tests. Conservatively speaking, a single block of activation and recovery (silent interval) can be achieved within 1.5–2 s.

### 4.3. MOCR Activity Was Measurable in All Participants

For any clinical test, it is vitally important that the test indeed measures the activity of the system it was designed to measure. In this vein, the fact that 100% of the participants had MOCR activation in at least one frequency (>90% participants across all seven frequencies) suggests that the approach and the parameters used in the present study is a feasible measure of MOCR function. More importantly, (1) these activations have passed a rigorous statistical test (HHG), and (2) display the characteristic time-course as reported by other human (Backus and Guinan, 2006) and animal studies (Liberman et al., 1996). As such, the certainty that these Δ over time are of MOCR in origin is higher than methods that reduce Δ to a single data point.

### 4.4. MEMR Influence

Inadvertent activation of the MEMR may negatively impact the confidence in MOCR estimation as both reflexes follow a similar time-course and have a similar impact on OAEs. Clinical tympanometry was used to indirectly determine the threshold of MEMR in most prior studies. More recent studies have consistently shown that this approach is not fail-safe (Guinan et al., 2003; Zhao and Dhar, 2009; Boothalingam and Goodman, 2021), as clinical tympanometers are relatively less sensitive and may underestimate MEMR thresholds by up to 20 dB (Feeney and Keefe, 1999; Feeney et al., 2003). There have been more recent efforts to detect MEMR presence in a more sensitive fashion using stimulus frequency emission group delay (Guinan et al., 2003; Zhao and Dhar, 2011), stimulus reflectance-based cut-offs (Abdala et al., 2014; Boothalingam and Purcell, 2015; Boothalingam et al., 2018), MEMR critical thresholds (Mertes, 2020) and using resampling techniques (Goodman et al., 2013; Mertes and Goodman, 2016; Lewis, 2017). Using the same time-course method used in this study, Boothalingam and Goodman (2021) showed that MEMR can be detected as stimulus level change with a high degree of certainty, based on its characteristic exponential growth. In addition, this approach is particularly useful for the present study because both the MEMR and the MOCR are elicited using the same stimulus.

A larger issue, however, is even if MEMR is detected, it cannot be ascertained if it will influence MOCR estimates. Our data (see Figure 4) suggests that even if MEMR is active it does not always necessitate influence on MOCR estimates. However, when the activation is large, in this case, >1 dB, there appears to be an influence on the MOCR estimate at 1 kHz. This is an important finding that may aid in the development of potential critical thresholds for MEMR influence on MOCR estimates. Critical thresholds can be useful in clinical settings, but they may not be universally valid. For instance, although Mertes (2020) established a statistical critical threshold for possible MEMR elicited using a 60 dB SPL noise elicitor, he indicated that critical thresholds can be influenced by a myriad of variables, e.g., choice of elicitor, elicitor level, OAE evoking stimulus, OAE evoking stimulus level, etc. Furthermore, critical thresholds suffer from the same issue, that it cannot be known if stimulus changes that breached critical threshold will affect MOCR estimation. One way around this problem is to run correlations between MEMR and MOCR estimates, as done in this study, to determine MEMR influence. This approach, however, is not feasible at an individual level. Therefore, each clinic should develop its own critical thresholds for their specific set of equipment and stimulus parameters.

The time-course method may offer a particular advantage over conventional methods in determining if MEMR activation influences MOCR. If the stimulus reflectance is reduced, the amount of stimulus energy reaching the cochlea is increased. As a result, the amount of MOCR activation will also be increased due to the increased stimulus energy, leading to a similarly larger CEOAE inhibition, Δ. In contrast, because the reflectance is relatively increased at higher frequencies (>~1.5 kHz), the stimulus reaching the cochlea is reduced. The amount of stimulus energy activating the MOCR is thus decreased, producing smaller CEOAE Δ. Notwithstanding the complications related to the interaction between CEOAE level, as a result of stimulus level changes due to variable reflectance, and MOCR inhibition of CEOAEs (Hood et al., 1996; Lewis, 2019), the time-course method may still be useful. This is because, while the size of Δ cannot distinguish the presence from the absence of MEMR activation, the direction of Δ change over time can. That is, at least at the higher frequencies where the stimulus reflectance increases over time, if the CEOAE Δ is completely driven by the MEMR, a similar increase in CEOAE Δ over time can be expected. Therefore, if we observe a CEOAE inhibition at these higher frequencies despite the increasing stimulus reflectance, an argument can be made that even if the MEMR was activated, it is not the predominant factor driving the CEOAE Δ over time. With an appropriate MEMR detection method and a critical threshold in place, MOCR estimates can be considered with more confidence. Furthermore, the results from our data suggest that despite relatively large MEMR activation in n13 at all frequencies, the influence on MOCR appears to be present only at 1 kHz. As such, it is possible that MEMR effects on MOCR is minimal at frequencies above 1.5 kHz. Further studies that use frequency-specific stimulation (e.g., tonebursts) may be able to shed further light on this conjecture.

## 5. Conclusion

We have introduced a time-course-based method of the MOCR magnitude (absolute Δ_*max*_) and kinetics estimated solely using clicks without any additional elicitors. The following highlights from our findings suggest that our proposed method can be successful in clinical translation. (1) 100% of the participants had MOCR activation in at least one frequency among seven 1/3rd bands (>90% across all seven frequencies). (2) The mean MOCR Δ_*max*_ during the bilateral activation (1.69 dB) is commensurate with the 1–2 dB OAE inhibition typically reported across MOCR studies using contralateral noise. (3) MOCR kinetics are commensurate with prior reports using SF- and DPOAEs (Kim et al., 2001; Backus and Guinan, 2006). (4) The higher-than-typical click level is advantageous in generating high SNR (Lewis, 2019). (5) Use of statistical tests allow for objective detection of MOCR activity. (6) The ability to concurrently detect for MEMR contamination allows for greater confidence in our results. Future studies that compare the method proposed here with conventional OAE-based MOCR methods in a within-subjects design are required to directly establish the benefits of the proposed approach.

## Data Availability Statement

The raw data supporting the conclusions of this article will be made available by the authors, without undue reservation.

## Ethics Statement

The studies involving human participants were reviewed and approved by Institutional Review Board, Northwestern University. The patients/participants provided their written informed consent to participate in this study.

## Author Contributions

SB and SD designed the experiment. HM collected the data. SB, SD, and SG analyzed the data and wrote the manuscript. All authors contributed to the article and approved the submitted version.

## Funding

This research was supported by an American Speech-Language-Hearing Foundation New Investigators Research Grant and the Office of the Vice-Chancellor for Research and Graduate Education, University of Wisconsin-Madison to SB and a Knowles Center Grant to SD.

## Conflict of Interest

The authors declare that the research was conducted in the absence of any commercial or financial relationships that could be construed as a potential conflict of interest.

## Publisher's Note

All claims expressed in this article are solely those of the authors and do not necessarily represent those of their affiliated organizations, or those of the publisher, the editors and the reviewers. Any product that may be evaluated in this article, or claim that may be made by its manufacturer, is not guaranteed or endorsed by the publisher.
